# Melioidosis in Thailand: Present and Future

**DOI:** 10.3390/tropicalmed3020038

**Published:** 2018-04-08

**Authors:** Soawapak Hinjoy, Viriya Hantrakun, Somkid Kongyu, Jedsada Kaewrakmuk, Tri Wangrangsimakul, Siroj Jitsuronk, Weerawut Saengchun, Saithip Bhengsri, Thantapat Akarachotpong, Somsak Thamthitiwat, Ornuma Sangwichian, Siriluck Anunnatsiri, Rasana W Sermswan, Ganjana Lertmemongkolchai, Chayada Sitthidet Tharinjaroen, Kanya Preechasuth, Ratchadaporn Udpaun, Poomin Chuensombut, Nisarat Waranyasirikul, Chanihcha Anudit, Surapong Narenpitak, Yaowaruk Jutrakul, Prapit Teparrukkul, Nittaya Teerawattanasook, Kittisak Thanvisej, Alisa Suphan, Punchawee Sukbut, Kritchavat Ploddi, Poolsri Sirichotirat, Bongkoch Chiewchanyon, Kamolchanok Rukseree, Maliwan Hongsuwan, Gumphol Wongsuwan, Pornpan Sunthornsut, Vanaporn Wuthiekanun, Sandy Sachaphimukh, Prapass Wannapinij, Wirongrong Chierakul, Claire Chewapreecha, Janjira Thaipadungpanit, Narisara Chantratita, Sunee Korbsrisate, Apichai Taunyok, Susanna Dunachie, Prasit Palittapongarnpim, Stitaya Sirisinha, Rungrueng Kitphati, Sopon Iamsirithaworn, Wipada Chaowagul, Ploenchan Chetchotisak, Toni Whistler, Surasakdi Wongratanacheewin, Direk Limmathurotsakul

**Affiliations:** 1 Bureau of Epidemiology, Department of Disease Control, Ministry of Public Health, Nonthaburi 11000, Thailand; soawapak@gmail.com (S.H.); skongyu@gmail.com (S.K.); 2 Mahidol-Oxford Tropical Medicine Research Unit, Faculty of Tropical Medicine, Mahidol University, Bangkok 10400, Thailand; viriya@tropmedres.ac (V.H.); tri@tropmedres.ac (T.W.); maliwan@tropmedres.ac (M.H.); gumphol@tropmedres.ac (G.W.); pornpan@tropmedres.ac (P.S.); lek@tropmedres.ac (V.W.); sandy@tropmedres.ac (S.S.); prapass@tropmedres.ac (P.W.); kae@tropmedres.ac (W.C.); cc12@sanger.ac.uk (C.C.); janjira@tropmedres.ac (J.T.); narisara@tropmedres.ac (N.C); 3 Faculty of Science, Prince of Songkla University, Songkla 90110, Thailand; jedsada.k@psu.ac.th; 4 Centre for Tropical Medicine and Global Health, Nuffield Department of Medicine, University of Oxford, Oxford, OX3 7FZ, UK; susie.dunachie@ndm.ox.ac.uk; 5 Faculty of Medicine, Prince of Songkla University, Songkla, 90110, Thailand; siroj.j@psu.ac.th; 6 Department of Clinical Pathology, Chiang Rai Prachanukroh Hospital, Chiang Rai 57000, Thailand; mistermummy9@gmail.com; 7 Division of Global Health Protection, Thailand Ministry of Public Health-US Centers for Disease Control and Prevention Collaboration, Nonthaburi 11000, Thailand; hpx4@cdc.gov (S.B.); hpt7@cdc.gov (T.A.); hpv2@cdc.gov (S.T.); xxc7@cdc.gov (O.S.); taw6@cdc.gov (T.W.); 8 Faculty of Medicine, Khon Kaen University, Khon Kaen 40002, Thailand; asiril@kku.ac.th (S.A.); rasanaw@gmail.com (R.W.S.); ploencha@kku.ac.th (P.C.); sura_wng@kku.ac.th (S.W.); 9 The Centre for Research & Development of Medical Diagnostic Laboratories, Faculty of Associated Medical Sciences, Khon Kaen University, Khon Kaen 40002, Thailand; g.lert@yahoo.co.uk; 10 Division of Clinical Microbiology, Department of Medical Technology, Faculty of Associated Medical Sciences, Chiang Mai University, Chiang Mai 50200, Thailand; chayada.si@cmu.ac.th (C.S.T.); kanya.p@cmu.ac.th (K.P.); ratchadaudp@hotmail.com (R.U.); 11 Department of Clinical Pathology, Chiangkham Hospital, Phayao, 56110 Thailand; chuensombut@hotmail.com; 12 Department of Clinical Pathology, Somdejphrajaotaksin Maharaj Hospital, Tak 63000, Thailand; Nisarat_mt@hotmail.com; 13 Department of Clinical Pathology, Uthai Thani Hospital, Uthai Thani 61000, Thailand; achanihcha@yahoo.com; 14 Department of Internal Medicine, Udon Thani Hospital, Udon Thani 41000, Thailand; Suraponguth@hotmail.com; 15 Department of Clinical Pathology, Udon Thani Hospital, Udon Thani 41000, Thailand; Yaowa_Ju@yahoo.co.th; 16 Department of Internal Medicine, Sunpasitthiprasong Hospital, Ubon Ratchathani 34000, Thailand; prapith1@hotmail.com (P.T.); vipada_1@yahoo.com (W.C); 17 Department of Clinical Pathology, Sunpasitthiprasong Hospital, Ubon Ratchathani 34000, Thailand; nidteeraw@gmail.com; 18 Department of Internal Medicine, Nakhon Panom Hospital, Nakhon Panom 48000, Thailand; kittisak97@gmail.com; 19 Ubon Ratchathani Provincial Public Health Office, Ubon Ratchathani 34000, Thailand; alisuphan@me.com; 20 Mukdahan Provincial Public Health Office, Mukdahan 49000, Thailand; mbdsmuk@yahoo.co.th; 21 The Office of Disease Prevention and Control 8, Udon Thani 41000, Thailand; dr.kritchavat@gmail.com; 22 The Office of Disease Prevention and Control 10, Ubon Ratchathani 34000, Thailand; bigtoii@yahoo.com; 23 The Office of Disease Prevention and Control 12, Songkla 90000, Thailand; b_chiewchanyon@yahoo.com; 24 Mahidol University Amnatcharoen Campus, Amnatcharoen 37000, Thailand; kamolchanok.ruk@mahidol.ac.th; 25 Department of Microbiology and Immunology, Faculty of Tropical Medicine, Mahidol University, Bangkok 10400, Thailand; 26 Department of Immunology, Faculty of Medicine Siriraj Hospital, Mahidol University, Bangkok 10700, Thailand; Sunee.kor@mahidol.ac.th; 27 Department of Infectious Diseases & Immunology, Emerging Pathogens Institute, University of Florida, Gainesville, FL 32611, USA; tuanyok@ufl.edu; 28 National Science and Technology Development Agency (NSTDA), Pathum Thani 12120, Thailand; prasit@nstda.or.th; 29 Department of Microbiology, Faculty of Science, Mahidol University, Bangkok 10400, Thailand; stitaya.sir@mahidol.ac.th; 30 Institute for Urban Disease Control and Prevention, Department of Disease Control, Ministry of Public Health, Bangkok 10220, Thailand; drrungrueng@hotmail.com; 31 Bureau of General Communicable Diseases, Department of Disease Control, Ministry of Public Health, Nonthaburi 11000, Thailand; sopon@ddc.mail.go.th

**Keywords:** *Burkholderia pseudomallei*, melioidosis, Thailand, mortality, diagnosis, surveillance, awareness, treatment, prevention

## Abstract

A recent modelling study estimated that there are 2800 deaths due to melioidosis in Thailand yearly. The Thailand Melioidosis Network (formed in 2012) has been working closely with the Ministry of Public Health (MoPH) to investigate and reduce the burden of this disease. Based on updated data, the incidence of melioidosis is still high in Northeast Thailand. More than 2000 culture-confirmed cases of melioidosis are diagnosed in general hospitals with microbiology laboratories in this region each year. The mortality rate is around 35%. Melioidosis is endemic throughout Thailand, but it is still not uncommon that microbiological facilities misidentify *Burkholderia pseudomallei* as a contaminant or another organism. Disease awareness is low, and people in rural areas neither wear boots nor boil water before drinking to protect themselves from acquiring *B. pseudomallei*. Previously, about 10 melioidosis deaths were formally reported to the National Notifiable Disease Surveillance System (Report 506) each year, thus limiting priority setting by the MoPH. In 2015, the formally reported number of melioidosis deaths rose to 112, solely because Sunpasithiprasong Hospital, Ubon Ratchathani province, reported its own data (*n* = 107). Melioidosis is truly an important cause of death in Thailand, and currently reported cases (Report 506) and cases diagnosed at research centers reflect the tip of the iceberg. Laboratory training and communication between clinicians and laboratory personnel are required to improve diagnosis and treatment of melioidosis countrywide. Implementation of rapid diagnostic tests, such as a lateral flow antigen detection assay, with high accuracy even in melioidosis-endemic countries such as Thailand, is critically needed. Reporting of all culture-confirmed melioidosis cases from every hospital with a microbiology laboratory, together with final outcome data, is mandated under the Communicable Diseases Act B.E.2558. By enforcing this legislation, the MoPH could raise the priority of this disease, and should consider implementing a campaign to raise awareness and melioidosis prevention countrywide.

## 1. Introduction

Indigenous melioidosis was first reported in Thailand by Chittivej et al. in 1955 [1]. The patient came from Saraburi Province, Central Thailand, presenting at Pramongkutklao Hospital, Bangkok, with lymphadenopathy. Pus collected from the patient was culture positive for *Burkholderia pseudomallei* [1]. In 1976, melioidosis was selected as the main discussion topic at the first meeting of the Infectious Disease Group of Thailand (now known as the Infectious Disease Association of Thailand) [2]. A total of 15 culture-confirmed melioidosis patients diagnosed in Bangkok up to 1976 was noted, and the clinical epidemiology of six fatal cases and two cases with unknown outcomes was discussed, raising the public health threat caused by melioidosis countrywide [2]. Subsequent workshops on melioidosis and interhospital case conferences organized by the Infectious Disease Group of Thailand and university hospitals increased the awareness of all physicians and laboratory staff of the need to look for melioidosis and *B. pseudomallei,* respectively, countrywide [3]. By 1985, 743 culture-confirmed cases of melioidosis had been reported from many provinces in Thailand; including Khon Kaen (*n* = 386; mortality 50%), Ubon Ratchathani (*n* = 169; mortality 61%), Bangkok (*n* = 91), Chiang Mai (*n* = 61), Nakhon Ratchasima (*n* = 30), Nonthaburi (*n* = 11), and Songkla (*n* = 6) [4].

In Thailand, underrecognition and misdiagnosis of melioidosis have been major obstacles to saving lives from this disease. Worldwide, untrained laboratory personnel commonly misidentify *B. pseudomallei* as a contaminant or other organism [5]. Leelarasamee et al. reported a total of 1165 isolates of *B. pseudomallei* identified in 46 hospitals from four regions of Thailand in 1995 (Northeast (*n* = 19), Central (*n* = 11), North (*n* = 9) and South (*n* = 7)) [6]. Nonetheless, the isolation rates were suspiciously low or absent in many hospitals when compared with adjacent hospitals that had reported very high numbers of melioidosis cases [6]. Focused training revealed that the microbiological laboratories in hospitals with unusually low numbers of melioidosis cases had misidentified the organisms, and the incidence rates of melioidosis in their areas were, in reality, also high [3]. 

From 1995 to 2015, melioidosis was heavily reported from Northeast Thailand [7,8] and rarely reported from the other regions. This led many clinicians and researchers to the misapprehension that melioidosis was not endemic in all regions of Thailand [9,10]. In this report, we review the latest information on melioidosis in Thailand and show that training in *B. pseudomalleii* identification for laboratory personnel is still needed countrywide.

The case fatality rate (CFR) of melioidosis in general hospitals in Thailand decreased markedly from 60–70% to 35–40% after the landmark study comparing ceftazidime with ‘conventional’ treatment was published in 1989 [11]. Unfortunately, in 2018—20 years later—the CFR of melioidosis in public hospitals is still around 30–35% [7,8]. Although the reported CFR is lower in university hospitals, at around 10–20% [9,12], most people in Thailand receive medical care in public hospitals, and university hospitals focus on tertiary care, providing service to less than 5% of the general population [13]. Generic ceftazidime is widely available, and the cost is covered by the universal coverage scheme; therefore, lack of effective antibiotics is not the issue in Thailand. To further reduce the CFR, it is likely that heavy investment in intensive care units, an increase in the ratio of nurses and doctors to patients, and an improvement in laboratory diagnostic capacity is needed. This has been done successfully in Australia, where the CFR of melioidosis is now approximately 10% [14,15,16,17]. For resource-limited countries such as Thailand, it might be better to invest in prevention campaigns and stop people from contracting melioidosis. However, the necessary developments leading to national prevention campaigns are impeded by the lack of disease awareness among lay people, lack of a national campaign by the MoPH to raise such awareness [18,19], and lack of official mortality data to support MoPH activities. 

It is still unknown how many patients die of melioidosis in Thailand yearly. A recent modeling study estimated that there are 7572 human melioidosis cases per year in Thailand, of whom 2838 (37%) die [8]. This is strongly supported by publications showing high numbers of culture-confirmed melioidosis cases diagnosed annually at major public hospitals in Thailand. Approximately 400 cases are diagnosed at Sunpasitthiprasong Hospital, Ubon Ratchathani, each year, of which about 150 result in death [7,20]. At Nakhon Panom Hospital, about 100 cases are diagnosed each year with about 30 deaths [21,22]. Melioidosis is one of the 83 diseases notifiable by law under the National Communicable Disease Surveillance system in Thailand. However, only 15, 10, 13, 4, and 12 deaths caused by melioidosis were officially reported to the Thailand disease surveillance system (Report 506) in 2010, 2011, 2012, 2013, and 2014, respectively [23,24,25,26,27]. It has been advised by the Bureau of Epidemiology, MoPH, that the total number of deaths based on Report 506 should not be used to represent the burden of the disease. However, these numbers have been used periodically by policymakers to represent the burden of melioidosis in Thailand to the public via the mass media (including newspapers and television broadcasts) [28]. Stakeholders occasionally compare the reported number of deaths due to melioidosis (for example, 12 deaths in 2014) with that of dengue (49 deaths in 2014) and leptospirosis (24 deaths in 2014) [28], and regard melioidosis as less important. Therefore, the lack of official melioidosis death reports via Report 506 hampers initiatives to improve awareness, diagnosis, treatment, and prevention of melioidosis in Thailand. 

The Thailand Melioidosis Network was formed in 2012, and has been working closely with the MoPH to investigate the burden, solve the problems of underreporting by Report 506, and develop initiatives on melioidosis. Here, we review the current guidelines for diagnosis, treatment and prevention of melioidosis in Thailand, explore how to improve the official data and increase awareness of melioidosis. We discuss the needs and future challenges to save lives from melioidosis in Thailand.

## 2. Melioidosis Cases and Presence of *B. pseudomallei* in Thailand 

**Figure 1:**
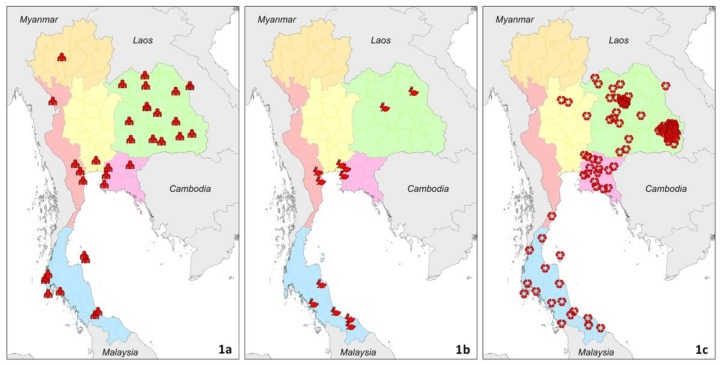
Evidence and distribution of melioidosis in Thailand from 1910 to 2015. Red icons represent geolocated records of culture-confirmed human cases (**1a**), culture-confirmed animal cases (**1b**) and presence of *B. pseudomallei* (**1c**). Green, orange, pink, rose, blue, and yellow colors represent Northeast, North, East, West, South, and Central Thailand, respectively. Interactive data are available online [54].

We reviewed evidence of melioidosis and presence of *B. pseudomallei* from 1910 to 2015 in Thailand (Figure 1). We searched PubMed for indigenous cases of melioidosis reported using the MeSH terms ‘melioidosis’ or ‘*pseudomallei*’ and ‘Thailand’. We also searched bibliographies from selected studies for secondary references. We included literature in English and Thai. Only culture-confirmed cases and cultures positive for *B. pseudomallei* from environmental samples were included. Clinically-suspected and serologically diagnosed melioidosis cases were excluded because of poor sensitivity and specificity of clinical manifestations and serological diagnostic tests for melioidosis [5]. We have described the evidence in each of six regions in Thailand: Northeast, North, East, West, South, and Central Thailand. This six-region division, developed by the National Geographic Committee in 1977, was used because it was set up for geographical and scientific purposes [29]. 

### 2.1. Northeast Thailand

Melioidosis is regarded as highly endemic in Northeast Thailand (Figure 1). It is the largest region of Thailand, and is divided into 20 provinces. This region is a plateau surrounded by mountain ranges, and much of the arable land consists of tropical sandy soil. Most of the published case reports and cohorts arise from four provinces: Khon Kaen, Nakhon Panom, Udon Thani, and Ubon Ratchathani. Srinagarind University, the Melioidosis Research Centre (MRC), Thailand MoPH–US Centers for Disease Control and Prevention Collaboration (TUC), and Mahidol Oxford Tropical Medicine Research Unit (MORU) have been collaboratively doing research with provincial hospitals in these four provinces for more than a decade. The published evidence demonstrates that melioidosis is highly endemic throughout the region. A study reported that 19 provincial hospital laboratories in Northeast Thailand diagnosed 1865 culture-confirmed cases of melioidosis in 2007 [7]. An environmental sampling study conducted in 2015 also found that *B. pseudomallei* is commonly present in six of the seven provinces evaluated, including Burirum, Chaiyaphum, Khon Kaen, Udon Thani, Nong Bua Lam Phu, and Loei, the exception being Nakhon Ratchasima [30]. We believe that the failure to detect *B. pseudomallei* in Nakhon Ratchasima is likely to be a false-negative result due to small sample size and poor sensitivity of the environmental sampling method [30], because *B. pseudomallei* is commonly found in clinical specimens in Nakhon Ratchasima Hospital [6]. The preliminary results of a large environmental study conducted by the Asia Partnership on Emerging Infectious Disease Research also found that *B. pseudomallei* was prevalent in fields on both sides of the main roads in Mukdahan province [31].

An increase in the total number of culture-confirmed cases diagnosed in Northeast Thailand, from 964 cases in 1994 (assuming one isolate per case) [6] to 1865 in 2007 [7], could be due to an increase in incidence rates of melioidosis, an increase in usage of bacterial culture as shown by the rise of the number of blood culture bottles used per year [32], and/or improved access to healthcare [33]. A genuine increase in incidence rates of melioidosis could be due to the rise in the prevalence of diabetes mellitus (an important risk factor for melioidosis) [34], the rising age of farmers in rural areas [35], increased land use for irrigated agriculture and rice farming [8], and/or depletion of soil nutrients caused by crop residue burning and poor farming practices [30]. The latter two points may also have led to an increase in presence of *B. pseudomallei* in the environment [8,30]. 

A number of captive zoo animals [36] and domestic animals [10,37] have been reported to have culture-confirmed melioidosis, both in Northeast Thailand and in all other regions in Thailand. A study by Kasantikul et al. on fatal culture-confirmed melioidosis in captive zoo animals in Thailand from 1997 to 2013 reported the highest incidence from the Northeast (*n* = 12) and South (*n* = 12) regions, followed by the East (*n* = 3), North (*n* = 1), and Central (*n* = 1) areas [36]. A descriptive study of culture-confirmed melioidosis in animals in Thailand from 2006–2015 by Kongkaew et al. also reported a total of 81 goats, 28 pigs, 18 cattle, 17 sheep, and 27 other livestock/pets at seven Veterinary Research and Development Center in six regions of Thailand [37].

### 2.2. North Thailand

North Thailand is composed of 9 provinces, some of which share a border with Myanmar and Laos. Its geography is comprised of several mountain ranges and the basins of four major rivers, which are tributaries of the Chao Phraya River in Central Thailand. Melioidosis has occasionally been reported from Chiang Mai, the largest province in the region [4,38,39]. From 2001 to 2003, 26 culture-confirmed cases were diagnosed at Maharaj Nakorn Chiang Mai Hospital, and 42% of them died [38]. A survey by Trakulsomboon et al. in 1999 reported that *B. pseudomallei* could be found in soil in North Thailand, but the details of locations were not reported, and an updated study has not been conducted [40].

In September 2017, a workshop organized by the MoPH, MRC, and Thailand Melioidosis Network was conducted at the Faculty of Associated Medical Sciences, Chiang Mai University, to provide information on melioidosis, the potential pitfalls in the identification of *B. pseudomallei* and diagnosis of melioidosis to laboratory staff from 17 hospitals in North Thailand and other regions. The immediate result was that Chiangkom Hospital, Phayao, North Thailand, diagnosed 20 culture-confirmed cases in 2017 [41]. During this workshop, we found that some provincial hospitals in North Thailand did not have high quality biochemical tests to identify *B. pseudomallei*, and misidentification as *Pseudomonas* spp. or contaminants was common. A recent study from Chiangrai Prachanukroh Hospital, Chiang Rai, also found that *B. pseudomallei* was commonly misidentified as *Acinetobacter* spp. [42]. Lack of laboratory staff training and the absence of an algorithm for identifying Gram-negative bacilli were likely to have been the main causes of the misidentification. In addition, the clinicians and laboratory staff were unaware that they were officially obliged to report culture-confirmed cases to the disease surveillance system (Report 506).

### 2.3. East Thailand

East Thailand is characterized by short mountain ranges alternating with alluvial plains. This region was historically not considered an endemic area for melioidosis; however, population-based surveillance activities by TUC in Sa Kaeo province, located on the Cambodian border about 200 kilometers from Bangkok, indicated otherwise [22]. Estimates from this project showed the average incidence of bacteremic melioidosis in Sa Kaeo from 2006–2008 to be 4.9 cases per 100,000 population (95% confidence interval (CI) = 3.9–6.1), with a population mortality rate of 1.9 per 100,000 (95% CI = 1.3–2.8) [22]. The annual incidence in Sa Kaeo was approximately 1/3 less than that determined for Nakhon Panom, an area considered highly endemic for *B. pseudomallei*, in this same study [22]. The CFR, however, was slightly higher in Sa Kaeo province at 44%, compared to Nakhon Panom province at 34% (*P* = 0.1) among patients with known outcomes from bacteremic melioidosis. These figures are likely to underestimate the true burden of *B. pseudomallei*, as the surveillance was focused on hospitalized cases. This study shows that the incidence of bacteremic melioidosis in Eastern Thailand is much higher than the figures reported to Report 506 of MoPH, Thailand suggest.

A descriptive study of animal melioidosis between 2006–2010 also reported a high number of culture-confirmed human melioidosis in two Eastern provinces, Chachoengsao (151 human cases and 2 goats), and Chonburi (76 human cases and 1 goat), where goat cases were diagnosed [10]. A recent environmental study also found *B. pseudomallei* to be highly prevalent in all six provinces (Sa Kaeo, Chachoengsao, Chonburi, Chanthaburi, Prachinburi, and Rayong) in East Thailand [30].

### 2.4. West Thailand

The geography of West Thailand is characterized by high mountains and steep river valleys along the border with Myanmar. Diagnosis of culture-confirmed melioidosis was rare within West Thailand until recently [10]. A descriptive study of melioidosis between 2006 and 2010 found a number of culture-confirmed human cases in two provinces, Ratchaburi (50 human cases and 16 goats) and Phetchaburi (8 human cases and 1 goat) [10]. A descriptive study of animal melioidosis by Kongkaew et al. also reported a high incidence of animal melioidosis in Ratchaburi, West Thailand, compared to all other provinces in the country [37]. 

During the workshop conducted at Chiang Mai University, North Thailand, in September 2017, laboratory staff from Somdejphrajaotaksin Maharaj Hospital, Tak, West Thailand, also participated. After the workshop, 3 culture-confirmed cases were diagnosed at the hospital that year [43]. TUC contacted Maesot General Hospital, Tak province, and noted that 15 bacteremic melioidosis patients had been diagnosed from 2013 to 2017. Finkelstein et al. also reported finding *B. pseudomallei* in soil samples collected from Prachuap Khiri Khan, West Thailand [44]. These results suggest that human melioidosis might have been misdiagnosed and underreported in West Thailand, which is on the border with Myanmar where melioidosis was first discovered in 1912 [45]. Training to improve diagnosis of melioidosis should include provincial hospitals in West Thailand.

### 2.5. South Thailand

South Thailand is famous for its beautiful beaches. The western part has steeper coastlines and is on the Andaman Sea, while the east side is mainly river plains and on the Gulf of Thailand. Melioidosis is increasingly recognized as endemic in South Thailand. One of the most striking phenomena was a number of culture-confirmed cases reported from tsunami survivors in 2004 in Phangnga [46,47,48], on the west coast of South Thailand. A foreign tourist was also suspected to have acquired melioidosis at Koh Samui, an island in Surat Thani on the east coast of South Thailand [49]. In 2012, an outbreak of 11 culture-confirmed cases (3 foreign tourists and 8 Thais) was observed in Koh Phangan, another island in Surat Thani on the east coast, and an environmental survey established that *B. pseudomallei* was widely found in water supplies on Koh Phangan [50]. Churangsuk et al. recently reported that, between 2002–2011, 134 culture-confirmed patients were diagnosed at Songklanagarind Hospital, Hat Yai, South Thailand [9]. Overall, 50% had localized infection, 37% were blood culture positive, and the CFR was 7% [9]. The low CFR is likely to be due to the high proportion of patients with localized infection [9], compared to cohorts diagnosed in other regions. Finkelstein et al. also reported the finding of *B. pseudomallei* in all 14 provinces in South Thailand [44]. Therefore, it is possible that melioidosis is endemic in all provinces, and further studies are needed to reveal the true burden of melioidosis in South Thailand. A training workshop was organized by MRC, Prince Songkla University (PSU), and Thailand Melioidosis Network during 20–21 December 2017 at PSU. A standard operating procedure (SOP) for laboratory diagnosis was provided, and laboratory staff from 6 provinces participated. The preliminary unofficial report from the office of Disease Control in Songkla showed a significant number of melioidosis patients, with high mortality [51].

### 2.6. Central Thailand

Central Thailand is a large plain consisting of clay soil. It includes 21 provinces and the Greater Bangkok area, and all published evidence of culture-confirmed human cases come from university hospitals in this area (Figure 1a). Most published cases report a history of travelling to Northeast Thailand, leading to the general understanding that those cases acquired *B. pseudomallei* outside the region, and that melioidosis is not endemic in Central Thailand. However, recent evidence in 2014 showed the presence of culture-confirmed melioidosis in ten goats in Bangkok, suggesting that *B. pseudomallei* might be covertly present in the environment in the central region and in Bangkok (Figure 1b) [52]. In addition, an environmental study published in 2016 conducted in seven provinces in central Thailand also confirmed the presence of *B. pseudomallei* in Phitsanulok, Phetchabun, and Nakonnayok (Figure 1c) [30]. It is likely that melioidosis cases are occurring in those three provinces, but they are possibly misdiagnosed and not reported. A recent animal study also found that both captive animals in a zoo and domestic animals had died of culture-confirmed melioidosis in Central Thailand [36,37]. During the workshop conducted at Chiang Mai University, laboratory staff from Uthai Thani, Central Thailand, also participated. In 2017, after the workshop, a total of 35 culture-confirmed cases were diagnosed at Uthai Thani hospitals [53]. These findings all strongly suggest that the common statement, ‘melioidosis is not endemic in Central Thailand’, is incorrect. 

Geographically, melioidosis is likely to be endemic throughout Thailand. Action is needed to evaluate whether *B. pseudomallei* isolated from clinical samples in all regions can be accurately identified by laboratory personnel in hospitals, whether melioidosis cases are diagnosed and treated correctly, and whether all culture-confirmed melioidosis cases observed in each hospital, including university hospitals, are reported to Report 506 accurately.

## 3. Current Recommendations and Availability of Measures against Melioidosis

Currently there are no official, national guidelines for diagnosis, treatment and prevention of melioidosis in Thailand. In general, the international consensus guidelines for diagnosis [5] and treatment [55] are used. 

Microbiological facilities are largely available in all provincial hospitals [32]. Following international sepsis campaigns [56], it is usual practice that doctors collect blood specimens for bacterial culture from every patient who presents at a provincial hospital and is prescribed parenteral antibiotics. In our experience, most Thai doctors do know about melioidosis and can diagnose and treat culture-confirmed cases of melioidosis appropriately if their laboratories can identify and report *B. pseudomallei* accurately.

However, there are no standard operating procedures (SOPs) or national guidelines for identification of *B. pseudomallei*. Most training and workshops are done ad hoc and do not include all microbiological laboratories in the country. The issue of misidentification of *B. pseudomallei* by many laboratories indicates that formal, national SOPs and training are critically needed to improve the identification of *B. pseudomallei*.

## 4. Surveillance Systems and Reporting of Melioidosis in Thailand

The National Communicable Disease Surveillance system (Report 506) was established in Thailand in 1968. Melioidosis has been one of the diseases for notification since 2002. However, until 2014, only about 10 fatal melioidosis cases per year were voluntarily reported in Report 506 [23,24,25,26,27]. The Thailand Melioidosis Network, together with the MoPH, has been investigating the issue of why provincial hospitals with microbiological facilities and fatal culture-confirmed melioidosis cases do not report them to the system. We found that many hospitals do not realize that they should report melioidosis cases, say that they are too busy to report them, or do not want to report a rapid increase of fatal melioidosis cases (for example, from 0 to 50 in a single province). We have been working through these issues since 2012, providing reassurance to hospitals that no negative consequences will result if the correct data are reported, that the MoPH is already aware of the situation, and that essential national action to reduce the deaths caused by melioidosis cannot be implemented if data are not reported. An improvement in reporting occurred in 2015. The number of melioidosis deaths formally reported rose to 112 in 2015 and 100 in 2016, solely because Sunpasithiprasong Hospital, Ubon Ratchathani province, began reporting (*n* = 107 and 87, respectively) [57,58]. It is likely that if all hospitals with microbiological laboratories in Thailand were to report the outcomes of culture-confirmed cases, the total number of deaths caused by melioidosis could be higher than 1000 cases per year [7]. 

Since June 2016, reporting of culture-confirmed melioidosis cases from every hospital with a microbiology laboratory, together with final outcome, has been mandated under the Communicable Diseases Act B.E.2558 (A.D. 2015) [59]. Melioidosis is now one of the 57 diseases with a legal requirement for notification [59]. It is, therefore, important to educate and remind laboratory staff, clinicians, statisticians, and epidemiologists to comply with the requirements of the existing surveillance system by including every single case of melioidosis with cultures positive for *B. pseudomallei* in their Report 506. By enforcing this legislation, the MoPH could raise the priority of the disease, and should also consider implementing a large campaign to raise awareness and implement melioidosis-prevention measures countrywide. 

We believe that the main cause for the discrepancy between the estimated number of fatal cases (*n* = 1000) and the model-predicted fatal cases (*n* = 2838) [8] is due to misdiagnosis and bacterial misidentification, which are still common outside Northeast Thailand.

In addition to the failure of reporting culture-confirmed cases, another important problem is the reporting of serologically diagnosed cases from community hospitals to the Report 506 system. Many of these cases are likely to be false positives, as it is well known that the indirect hemagglutination assay (IHA) should not be used to diagnose melioidosis in endemic countries due to the high background seropositivity [5]. This has led to a high number of false-positive melioidosis cases reported to Report 506, with few deaths, and therefore, reported CFRs are very low. For example, the CFR of melioidosis was officially reported as 0.5% (15/2902), 0.3% (10/3920), 0.2% (13/3711), 0.1% (4/2836), and 0.2% (12/2544) for Report 506 in 2010, 2011, 2012, 2013, and 2014, respectively [23,24,25,26,27]. These incorrect CFRs (ranging from 0.1 to 0.5%) have led to a false sense of security. People and policymakers mistakenly believe that melioidosis is not an important infectious disease in Thailand, as less than 1% of reported melioidosis cases were reported to have died. This is highly inaccurate, as the observed CFR of culture-confirmed cases in correctly designed studies in Thailand is between 30–35% [7,8].

The Thailand Melioidosis Network has proposed revising the reporting system to cover only culture-confirmed cases so that the true burden and true mortality (and CFR) could be demonstrated to the MoPH. Nonetheless, due to the problem of maintaining diagnostic capacity for melioidosis at community hospitals, the proposal was not accepted. The community hospitals require affordable rapid diagnostic tests (RDT) with high accuracy (particularly with high positive predictive value in melioidosis-endemic countries) to justify replacement of the IHA. Therefore, such an RDT is critically needed for Thailand.

Currently, the MoPH is working to rectify the underreporting of deaths caused by melioidosis in Report 506 by using (1) the hospital database known as ‘43 files reports’ and (2) existing microbiological databases. The 43 file reports include International Statistical Classification of Diseases 10 (ICD-10). According to ICD-10, melioidosis is coded as A24. The MoPH is evaluating how many patients with the ICD-10 code of A24 were reported to Report 506, and how many of those died countrywide. Other work has used laboratory microbiological databases, and will evaluate how many patients with cultures positive for *B. pseudomallei* were reported to Report 506, and how many of those died countrywide. These activities will support the MoPH to improve understanding of the burden of melioidosis and the discrepancies between cases diagnosed by attending physicians, microbiologically-confirmed cases, and those reported to Report 506.

## 5. Awareness of Melioidosis in Thailand

Awareness of melioidosis in the general Thai population is very low. A recent study showed that 74% of lay adults had never heard of melioidosis, and 19% had heard of the disease, but had no further knowledge [18]. Information about melioidosis is rarely given to the public by the mass media, including television, newspapers, and radio stations. Basic information about the disease and its prevention is also not taught in schools in Thailand [18]. In contrast, public awareness of other common infections, such as HIV/AIDS, tuberculosis, malaria, leptospirosis, dengue, and influenza, are high, as these topics are taught in schools and are frequently mentioned by the national media [18]. 

A contributing factor to poor awareness of the disease is that many melioidosis patients die quickly before the microbiological results have been reported to the doctors caring for the patient. Even for culture-confirmed cases of melioidosis, doctors and healthcare workers are often too busy to explain to patients and their families what melioidosis is. This is because the relatives have usually never heard of this disease, and the explanation would take time. Most relatives in such scenarios are informed that the cause of death was sepsis, septic shock, or bacteremia, without explaining about melioidosis. They are also not informed about how to prevent other people in the family, or in the community, from acquiring the same disease. Although those who survive melioidosis are educated about the disease and receive oral eradicative treatment for up to 20 weeks, knowledge about the importance of the disease is not effectively shared with their relatives, friends, or communities. This is shown by the fact that most have never heard of the disease.

## 6. Major Changes and/or Achievements

The Thailand Melioidosis Network was formed during a meeting in March 2012 [60]. The meeting included scientists conducting melioidosis research and policymakers from across Thailand. The main purpose was to (1) provide updated information to participants, (2) form collaborations among research institutes and government authorities, and (3) discuss the important issues related to melioidosis in Thailand, including epidemiology, diagnosis, treatment, prevention, public awareness, and public engagement. The second meeting was held in September 2012 [61]. After the first two meetings, group email was used to communicate among the members.

The first international congress devoted to melioidosis held in Thailand, entitled ‘International Congress on Melioidosis’ with the theme ‘State of the art discoveries and trends towards the 21st century’, took place at the Siam City Hotel in Bangkok in 1998. The congress was organized by the Chulabhorn Research Institute and the chairperson was Prof. StitayaSirisinha. There were 150 delegates from Thailand and several other countries. The meeting was later referred to as ‘The 2nd World Melioidosis Congress’, which has since been held every three years and rotated through melioidosis-endemic countries. The international melioidosis meeting held in Kuala Lumpur, Malaysia in 1994 and chaired by Prof. Savithiri Puthucheary was regarded as the first World Melioidosis Congress (WMC). The 3rd and 4th WMC were held in 2001 in Perth, Australia, and 2004 in Singapore, respectively. In 2007, the 5th World Melioidosis Congress was again held in Thailand at Khon Kaen province and hosted by MRC, with Prof. Surasak Wongratanacheewin as chairperson [62], followed by the 6th WMC in Townsville, Australia. In 2013, over 300 delegates from 24 countries attended the 7th WMC in Bangkok [63]. It was hosted by MORU and the Faculty of Tropical Medicine, Mahidol University, with Dr. Wirongrong Chierakul as chairperson. The 8th WMC was hosted by University of Florida in Cebu, Philippines in 2016, with Asst. Prof. Apichai Tuanyok and Prof. Herbert Schweizer as chairpersons.

Hosting these meetings in Thailand has helped to raise the profile of melioidosis in the Thai medical, scientific, and political communities, as well as attracting media attention for the general public. 

## 7. Current and Future Challenges

During the meetings of the Thailand Melioidosis Network in 2012, four priority areas were identified [61]. These included (1) to enhance the surveillance system, (2) to increase public awareness, (3) to improve diagnosis of melioidosis, and (4) to improve prevention of melioidosis. 

First, the problem of underreporting by Report 506 must be solved. Although the rise in the reported number of deaths in 2015 occurred, more needs to be done. Only one provincial hospital (Sunpasitthiprasong Hospital) reported its melioidosis cases and their mortalities to Report 506. This was partially supported by the research unit in Sunpasitthiprasong Hospital. In future, the personnel who are responsible for the system might change jobs, and the system is not automated. Although a lot of discussion with many hospitals has taken place, the data from these hospitals have not yet been submitted to the national reporting system. An immediate need is educating and supporting all hospitals with microbiological facilities to report their culture-confirmed melioidosis cases and their mortalities to Report 506. A long-term solution is to automate the reporting system, similar to the systems in the Netherlands and China [64]. Another option is to have all admission and laboratory data from all hospitals in Thailand collected into one central data system. Then, formal analysis could be performed to identify the total number of cases for the Annual Epidemiological Surveillance Report of the MoPH [65], rather than using data from Report 506 alone. 

Secondly, in order to raise public awareness of melioidosis, a large national campaign led by the MoPH is needed. Based on multiple studies in Thailand [18,19], lay people doubt that melioidosis is responsible for more than 1000 deaths of Thai people each year. They often ask, if that were true, “Why have I never heard or seen anything about this disease on television or in any campaign in hospitals?” Credible sources are a crucial component to provide information about the disease [18,19]. Thai people rely on doctors, nurses, healthcare workers, and the government to provide regular information about diseases and their prevention [18,19]. Therefore, information must come from the MoPH on a scale that is appropriate to a disease that kills more than 1000 people each year and is preventable. If people do not know about the disease and its true fatality rate, then prevention will be nearly impossible [18,19]. 

Third, the problem of misdiagnosis and bacterial misidentification in hospital microbiological laboratories outside Northeast Thailand has been described above. This is very likely due to the fact that (1) most laboratory technicians erroneously believe that melioidosis is endemic only in Northeast Thailand, (2) laboratory training around diagnostic testing is not adequate, (3) there is a lack of rapid bacterial identification tests, along with poor usage of *B. pseudomallei* selective culture media, and (4) a lack of RDTs for melioidosis diagnosis direct from clinical specimens, obviating the need for bacterial culture. Major university hospitals in Thailand could use instruments such as the Vitek 2 system (BioMerieux, Lombard, IL, USA) or matrix-assisted laser desorption ionization-time of flight mass spectrometry (MALDI-TOFMS), if the appropriate databases for *B. pseudomallei* are available [66]. However, it is crucial that all microbiology laboratory personnel in Thailand should be sufficiently familiar with *B. pseudomallei* to identify the organism within available resources, for example, using standard biochemistry and drug susceptibility tests. All technicians should be trained to recognize *B. pseudomallei* colonies, and not discard them as contaminants. All oxidase-positive Gram-negative bacilli should be tested to see whether they are *B. pseudomallei* [5]. Reports of *Pseudomonas* spp. should include a note that the organism was tested and confirmed not to be *B. pseudomallei*. A simple three-disc susceptibility testing method (demonstrating resistance to gentamicin and colistin but sensitivity to amoxicillin/clavulanic acid [67,68]) should be used in areas where resources are limited and secondary diagnostic tests (such as API 20NE and latex agglutination specific for *B. pseudomallei*) are not available. All uncertain isolates should be sent for confirmation to the Department of Medical Science, MoPH, Thailand, university hospital laboratories, or any research organizations in the area willing to receive bacterial isolates for confirmation of identity. 

A latex agglutination assay using a monoclonal antibody specific for *B. pseudomallei* has been available in Thai research organizations for more than 17 years [69]. The test is useful for isolate confirmation, but cannot be used directly on clinical specimens. The Thailand Melioidosis Network strongly advocates for the latex agglutination assay to be one of the standard tests available to all microbiological laboratories in Thailand, together with the appropriate training. This should help to solve the problem of misidentification of *B. pseudomallei* countrywide [61].

A lateral flow immunoassay (LFI) using a similar monoclonal antibody to the latex agglutination assay has been developed by InBIOS (Seattle, WA, USA), and is currently being evaluated in several countries [70,71]. LFI can be used for rapid colony identification in a similar way to latex agglutination, and has the potential for application directly on clinical specimens [70,71]. Other advantages are lower cost when economy of scale is high, and longer shelf life. Currently, Prof. Wongratanacheewin at MRC is funded by the National Science and Technology Development Agency of Thailand, to develop a LFI for use in Thailand. The LFI could eventually replace latex agglutination as the production costs would likely be lower, and it should easily be manufactured at adequate levels to supply the whole country.

Fourth, recommendations for melioidosis prevention should be promoted thoroughly and intensively. Thailand is a hotspot for melioidosis, but only a small proportion of people in Thailand follow recommended behaviors that can prevent melioidosis [18,19]. These include using protective gear, such as rubber boots and gloves when in direct contact with soil and surface water, and consuming bottled or boiled water. However, changing behavior is typically complex, and providing information and boots alone is unlikely to be effective. This is shown by the fact that boots have been provided to Thai farmers for leptospirosis prevention since 2000 [72], yet many people still work in rice fields without using them [73]. Recent studies found that Thai people had no knowledge of melioidosis, believed that there was no need to adopt the recommended preventive behaviors, and were not inclined to use boots and gloves while working in muddy rice fields [18,19]. Practically, over-the-knee boots can be used in flooded rice fields without causing difficulty in walking, but they are still uncomfortable to wear in hot weather. Participants reported that input from numerous role models (physicians, diabetic clinics, friends, and families), and from the government via mass media, would be required for them to change their behavior. We strongly recommend that a multifaceted intervention at community and government levels is required to bring about the desired changes. It is still unknown how many melioidosis cases could be prevented by each recommended behavior. The size of the effect and cost-effectiveness of those preventive measures is being evaluated in a large behavioral change trial (NCT02089152). Nonetheless, the MoPH need not wait for the final results of this study (expected at the end of 2019) before launching a campaign, because the recommended preventive measures are simple and align with those for many other infectious diseases. The recommendations are supported by epidemiological studies [73,74], and are similar to prevention campaigns to raise awareness and reduce the number of cases and deaths due to melioidosis that have been conducted for several years in northern Australia [75].

In the long term, an effective vaccine against melioidosis, targeting at-risk groups, such as people with diabetes and agriculture workers, would be the ideal strategy for reducing the burden of disease and number of deaths from melioidosis in Thailand [76], and could be cost effective [77]. Ongoing research programs in Thailand [78] and internationally [79,80] to identify vaccine candidates are making progress, but vaccine development needs a long timeframe, and the case for ongoing funding of vaccine research relies on demonstrating the true extent of the disease burden in countries like Thailand.

In conclusion, we strongly believe that a prevention campaign should be undertaken now by the MoPH, together with enhancing disease surveillance systems, raising awareness of the disease and its true mortality, and improving the diagnosis of melioidosis. We believe that formal campaigns from the MoPH would enhance the usage of free boots that MoPH regularly provides to farmers. More lives could be saved, not only from leptospirosis, but also from melioidosis.
